# Missed Massive Pulmonary Embolism and the Hidden Threat of a Distal Deep Vein Thrombosis

**DOI:** 10.1177/2324709617754117

**Published:** 2018-01-31

**Authors:** Cynthia Wong

**Affiliations:** 1 Northeast Lincolnshire and Goole NHS Trust, Grimsby, UK

**Keywords:** pulmonary embolism, deep vein thrombosis, venous thromboembolism

## Abstract

A 47-year-old woman presented to her GP (general practitioner) surgery with a left leg pain of 4 days duration after a recent 4-hour flight. She was taking the oral combined contraceptive pill and had no past medical history. She had a low predictive Wells score for deep vein thrombosis, but her D-dimer was positive, so she had a proximal lower limb vein ultrasound scan as per the National Institute for Clinical Excellence guidelines, which was negative. Two days later, she presented to the emergency department with a collapse and dyspnea. Her blood pressure was unrecordable in the ambulance, and she was severely peripherally cyanosed with a blood pressure of 64/40 mm Hg in the emergency department. She had a computed tomography pulmonary angiogram, which confirmed extensive bilateral pulmonary emboli with right ventricular strain. She had 2 cardiac arrests while in hospital and is now on long-term anticoagulation.

## Introduction

A pulmonary embolism (PE) is a potentially life-threatening venous thromboembolism (VTE) that requires immediate management. Many PEs tend to stem from a deep vein thrombosis (DVT), so DVTs need to be treated promptly to prevent serious complications. The lifetime risk of a VTE is very high at approximately 100 persons per 100 000 each year.^1^ The current National Institute for Clinical Excellence (NICE) guidelines state that a DVT with an unlikely 2-level Wells score should have a D-dimer test done. A positive D-dimer indicates that a proximal leg vein ultrasound scan should be arranged within 4 hours. Those with a negative ultrasound scan and a positive D-dimer test should have a repeat within 6 to 8 days.^2^

This case involved a patient who presented with an unlikely DVT and was appropriately managed as per the NICE guidelines. However, she subsequently developed very severe complications that suggest that the current guidelines were not appropriate in this case.

## Case Report

A 47-year-old woman presented to her GP (general practitioner) surgery with a left leg pain of 4 days duration. She described the pain as a relatively painful ache, which started not long after her flight from Crete back to England 5 days ago. She had also injured her leg slightly by tripping while she was in Crete, which had caused a bit of pain at the time. She was otherwise well with no cough, shortness of breath, chest pain, or hemoptysis.

She had no significant past medical history. She was taking the combined oral contraceptive pill. She was a nonsmoker. She had no significant family history.

**Figure 1.**
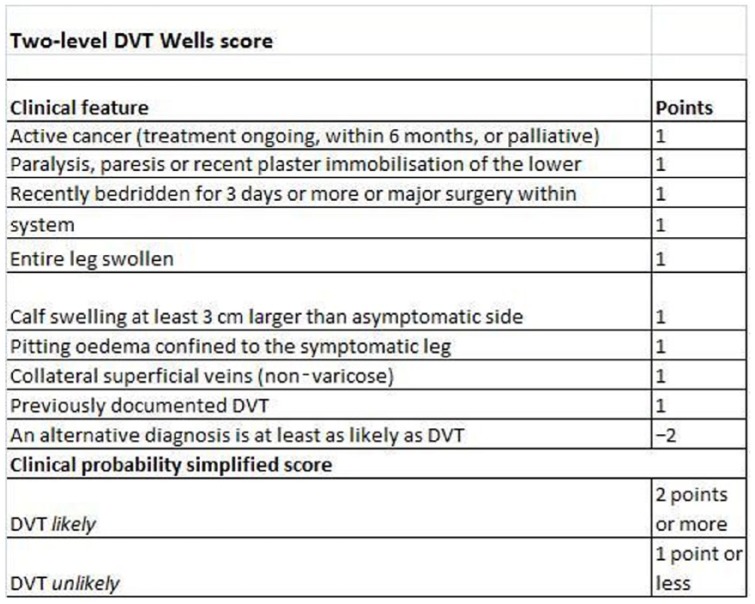
Modified Wells clinical probability score of DVT (deep vein thrombosis). Reproduced from NICE (National Institute for Clinical Excellence), adapted from Wells et al.^5^

Her vital signs were stable with a heart rate of 75 beats per minute, a blood pressure of 128/87 mm Hg, and respiratory rate of 12 breaths per minute. She was of normal body habitus, including a body mass index of 23 kg/m^2^, and appeared otherwise well besides the leg pain. Modified Wells score was −1, with less than 3 cm discrepancy between the size of the legs, no varicosities, no previous history of DVTs, no history of malignancy, and no recent immobilization (see Figure 1).

**Figure 2.**
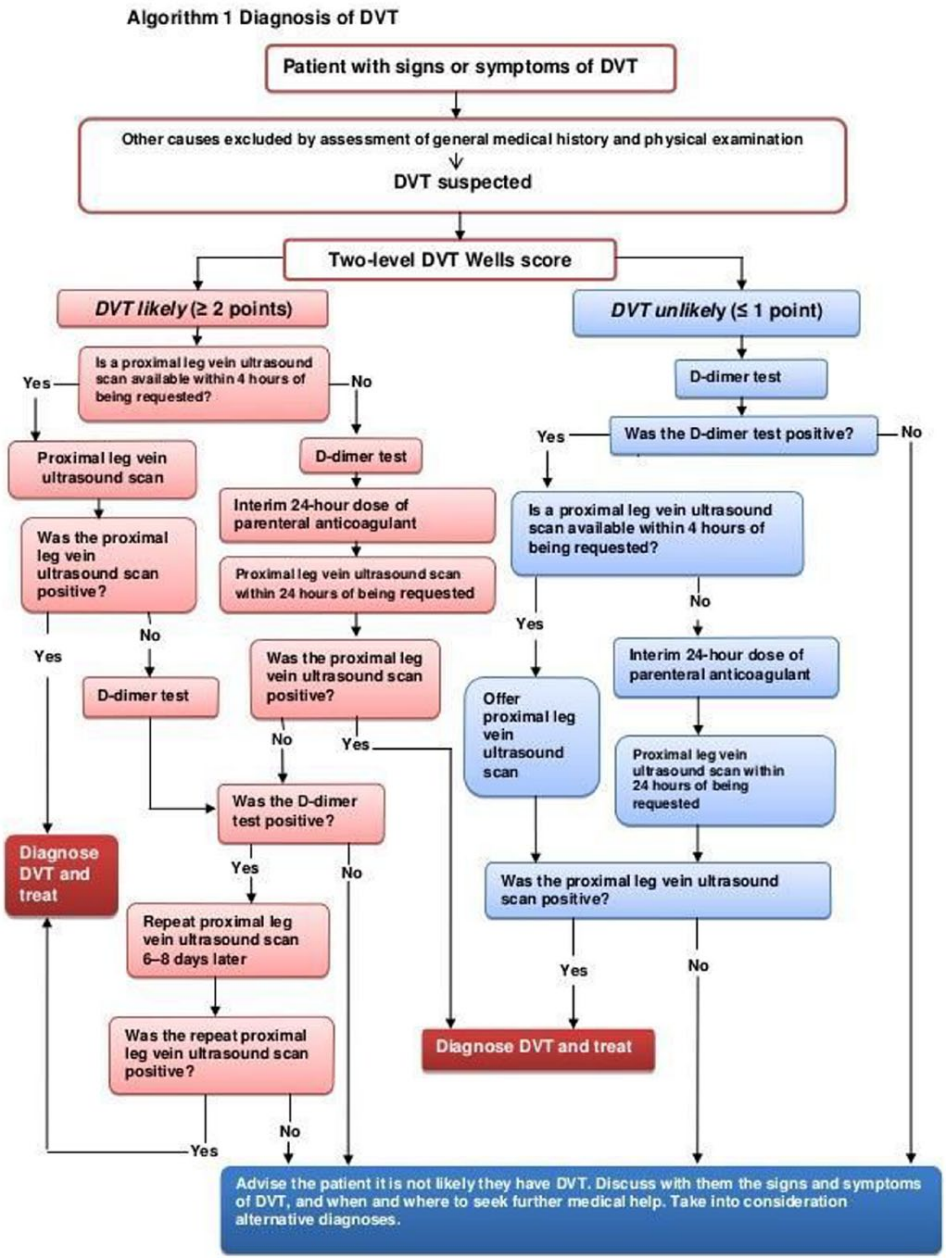
Clinical algorithm for diagnosis of DVT (deep vein thrombosis). Reproduced from NICE (National Institute for Clinical Excellence).

Although the Wells score was low, she had a point of care D-dimer performed, which was 4.0 µg/mL (reference range for upper limit of normal is <0.46). In view of this raised D-dimer, a Doppler ultrasound at the hospital was arranged urgently as per the protocol for DVTs (see Figure 2). She was found to be negative for a DVT after scanning the proximal left lower extremity.

The differential diagnosis at this point included a muscle sprain and a DVT. In view of the negative ultrasound, she was treated conservatively as a muscle sprain and advised to return urgently if her pain persisted over the weekend (she had initially presented on a Thursday, was scanned the same day, and reviewed on the Friday), to return for further review on Monday. This was in line with the guidelines, which suggested that a repeat ultrasound could be performed in a week’s time if the initial ultrasound was negative despite positive D-dimer (see Figure 2).

Two days later, she presented to the emergency department with a collapse and dyspnea. Her blood pressure was unrecordable in the ambulance, and she was severely peripherally cyanosed with a blood pressure of 64/40 mm Hg in the emergency department. Her heart rate was 54 beats per minute and pulse oximetry was unrecordable. She had an urgent computed tomography pulmonary angiogram, which confirmed extensive bilateral pulmonary emboli, central and segmental, with right ventricular strain. She was transferred back to the resuscitation unit and arrested with pulseless electrical activity and then ventricular fibrillation. She was intubated during the arrest.

She was subsequently transferred to the intensive care unit and was commenced on heparin infusion. She had a second cardiac arrest in the intensive care unit with pulseless electrical activity and was resuscitated successfully and continued with heparin, as she was unstable for surgical intervention at the time.

Bedside transthoracic echocardiogram confirmed impaired right ventricular function. She underwent thrombectomy the following day in view of her rising lactate and poor response to inotropes and thrombolysis. Because of her ongoing anemia despite transfusion, she had a computed tomography scan of the thorax, abdomen, and pelvis, which did not show any bleeding source. Her renal function deteriorated and she was commenced on temporary hemodialysis. Investigation into hypercoagulable disorders was negative, with factor V Leiden, prothrombin 20210, antithrombin, protein C, protein S, immunoglobulins, complement C3/C4, monoclonal protein, serum paraprotein, MPO/PR3, anti-cardiolipin, ANA, β_2_ glycoprotein, dsDNA, and ENA screen all negative.

She is currently continuing anticoagulation (having been discharged on enoxaparin) and will be followed-up by the respiratory and renal team, as well as repeating the transthoracic echocardiogram in 3 months’ time.

## Discussion

Based on the modified Wells score (see Figure 1), this patient was assessed as low risk and managed appropriately; however, this case highlights how the Wells score fails to take into account risk factors that are commonly known to increase the risk of VTE. In this case, the patient’s recent flight and use of the combined oral contraceptive pill were major risk factors, with evidence suggesting that air travel doubles the risk of VTE, and when combined with oral contraceptive use, ends up increasing the risk by 14-fold.^3,4^ Rather than the current narrow criteria, a wider range of prothrombotic factors should be considered within the scoring system. The modified Wells score has limitations and even patients who are a predicted low probability have 5% prevalence of DVT.^5^

**Figure 3.**
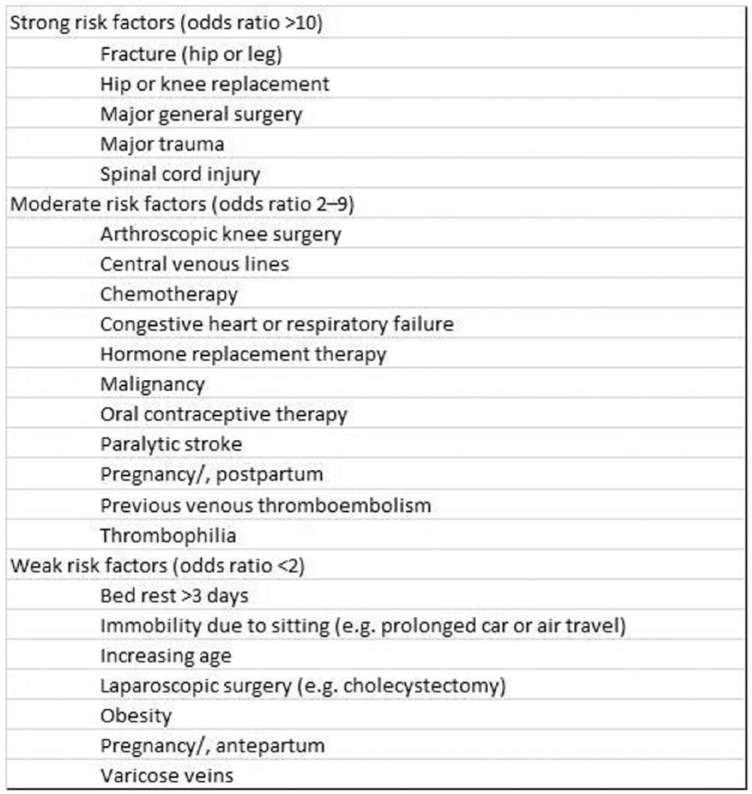
Risk factors for VTE (venous thromboembolism). Adapted from Anderson et al.^6^

Any scoring system that is used to determine probability of VTE should include more of the relevant risk factors. It is thought that 75% to 96% of patients with a VTE will have at least one risk factors (see Figure 3).^6^ Also of note is that the Wells score does not take into account the different gradients in severity of VTE risk factor, instead equating all the included risk factors as similarly influential to the overall score. However, Wells is a well-validated score, while the evidence supporting air travel as a risk for developing VTE is based on lower quality evidence and it has been difficult to draw definitive conclusions.^5,7^

Considering the patient initially presented with pain in her calf, it seems reasonable to assume that the DVT was limited to the distal segment of the lower limb in view of the negative proximal ultrasound scan, although these tests have false negative rates. Distal ultrasound scans are not performed by sonographers largely because of lack of training, as well lower sensitivity and specificity for calf DVT, though studies do suggest with the appropriate training, it can be considered a valid and feasible test,^8^ but it may be reasonable to make an alteration to the guidelines in view of the severe complications that arose from a distal DVT in this case. This alteration may require more invasive investigations such as contrast venography or a lower threshold for starting anticoagulation if the D-dimer is raised, even if the proximal ultrasound was clear.^9^

Although the NICE guidelines suggest follow-up in 6 to 8 days with a further ultrasound scan, this case illustrates how this suggested time span may be too long, as this patient developed a large PE within 2 days.^2^ Based on this case, it may be beneficial to consider rescanning earlier if a patient has a negative initial ultrasound but a positive D-dimer to confirm that there is no evolving VTE.

This case highlights how the Wells score for assessing probability of DVT does not take into account many situations where a patient would be in a hypercoagulable state. Particular consideration should be given to the combination of air travel in combination with oral contraceptive use as a risk factor for VTE. The current NICE guidelines for diagnosing a DVT specifies that proximal ultrasound scans should be obtained, which fails to catch any distal DVT. Even a distal DVT can have severe, life-threatening complications and should be managed similarly to a proximal DVT. A repeat leg vein ultrasound should be performed after the initial scan to rule out any evolving clot if the D-dimer is positive.
